# Woven-like change following intracoronary thrombosis recanalization: a case report

**DOI:** 10.1186/s12872-019-01283-5

**Published:** 2019-12-30

**Authors:** Wei Wen, Haibo Liu, Jimin Li, Qi Zhang

**Affiliations:** Department of Cardiovascular Medicine, Shanghai East Hospital, Tongji University School of Medicine, Shanghai, China

**Keywords:** Woven coronary artery, Thrombosis recanalization, Percutaneous coronary intervention, Intracoronary image

## Abstract

**Background:**

A woven coronary artery is a rare congenital coronary anomaly and incidentally found in coronary angiogram. Coronary angiography is the major diagnostic modality, which shows the main trunk of coronary divides into several channels which later reconnect with normal blood flow (J Int Cardiol 113:121-123 2006). However, some cases and reviews inferred that this characteristic might be mimicked by recanalized coronary thrombus, which occurs following thrombotic occlusion. In some case, the multiple intraluminal channels have a ‘Swiss cheese’, a ‘Spider web-like’, a ‘Honeycomb’ or a ‘Lotus root’ appearance and most of them appear in local segment (Int J Cardiol 186: 239–240, 2015). As these images are nonspecific findings, there is no angiographic uniform definition of intracoronary thrombus. More information about the characteristics and the development of this woven-like structure is needed.

**Case presentation:**

A 67-year-old male patient was admitted to our hospital because of chest pain. Coronary artery angiogram revealed that the right coronary artery (RCA) divided into multiple thin channels from proximal to distal, which was similar to the so-called woven coronary artery. Compared with his prior coronary angiograms which showed a tiny hazy lesion in distal segment of RCA, we found the woven-like phenomena should be caused by a late-stage recanalized coronary thrombus. Percutaneous coronary intervention (PCI) was performed to restore the RCA flow, and the angina symptom was extremely improved during clinical follow-up.

**Conclusions:**

The diagnostic criteria of woven coronary artery was based on angiographic image. However, some cases and reviews inferred that thrombotic recanalization might also share the same characteristic. In this case, we collected the baseline angiograms and intracoronary images then successfully diagnosed the woven-like RCA as thrombotic recanalization. For this kind of woven-like coronary artery, PCI could be a better treatment strategy. Detailed history collection and intracoronary image techniques should be emphasized in future clinical practice in the differentiating and treatment of woven-like phenomena.

## Background

Woven coronary artery was described by Sane for the first time in 1988 [1]. It is considered as a rare congenital coronary anomaly and incidentally identified in coronary angiogram. Individual case reports of woven coronary artery have been published so far, and now the diagnostic criteria is based on angiographic image. However, it was mentioned that this angiographic characteristic might be mimicked by thrombotic recanalization, which is a rarely recognized entity after thrombotic occlusion. Differential diagnosis of such woven-like images was very important for the treatment strategy.

## Case presentation

A 67-year-old male patient was admitted to our hospital for chest pain in August 2018. The electrocardiograph showed atrial fibrillation in rhythm and Q wave in the inferior leads. Transthoracic echocardiography showed inferior wall with decreased left ventricular ejection fraction (LVEF) of 40%. The patient was admitted in hospital 6 years ago. Unluckily, all the information was untraceable. Coronary angiography (CAG) was suggested and performed on 31st July 2018. We found that his main trunk of RCA was divided into multiple twisting small channels from proximal to distal, which reconnected at the level of posterior descending artery (PDA) with normal blood flow, the posterior lateral branch (PLB) was totally occluded and supplied by contra-lateral collaterals. The LCA was normal (Fig. 2 a-b). This patient was firstly diagnosed with woven coronary artery because of the angiographic characteristic.

**Fig. 1:**
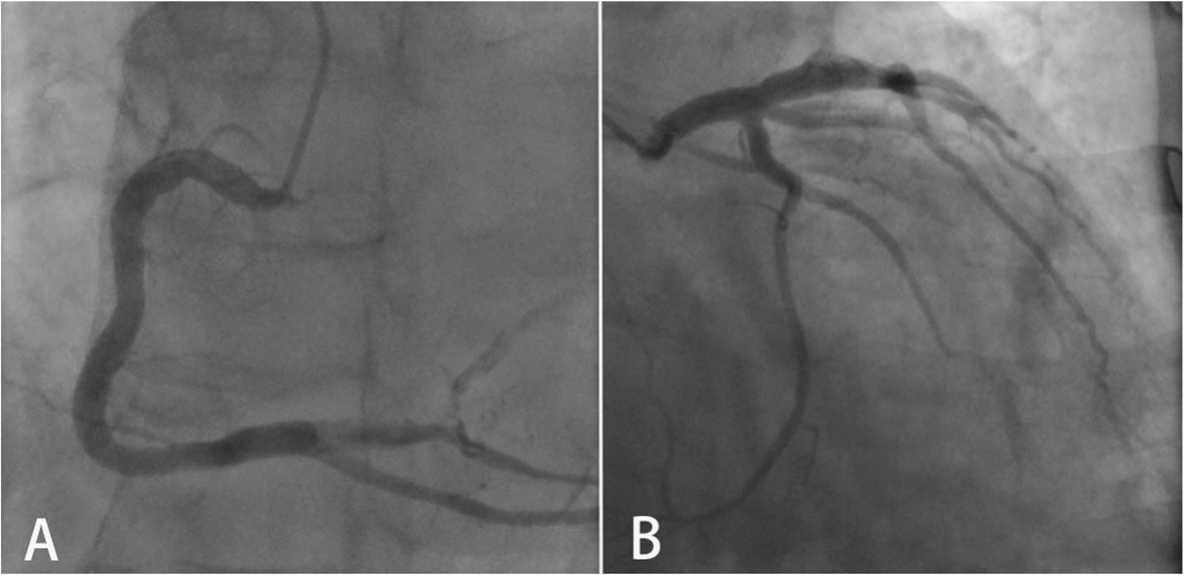
The CAG data on 12th July 2012. CAG in July 2012 showed that the RCA was patent, thrombus containing lesion was existed in distal segment TIMI flow 2 (**a**). The LCA was normal (**b**)

After we traced this patient’s prior CAG data on 12th July 2012, the angiogram showed a tiny hazy lesion with an 80% stenosis in the distal segment of the RCA, which was treated medically based on the patient’s willing, and his left coronary artery (LCA) was normal (Fig. 1).

Based on these findings, we surmised that the woven-like change was caused by the progression, organization and re-canalization of thrombus. The program of PCI for the RCA was implemented on 2nd Aug 2018.

**Fig. 2:**
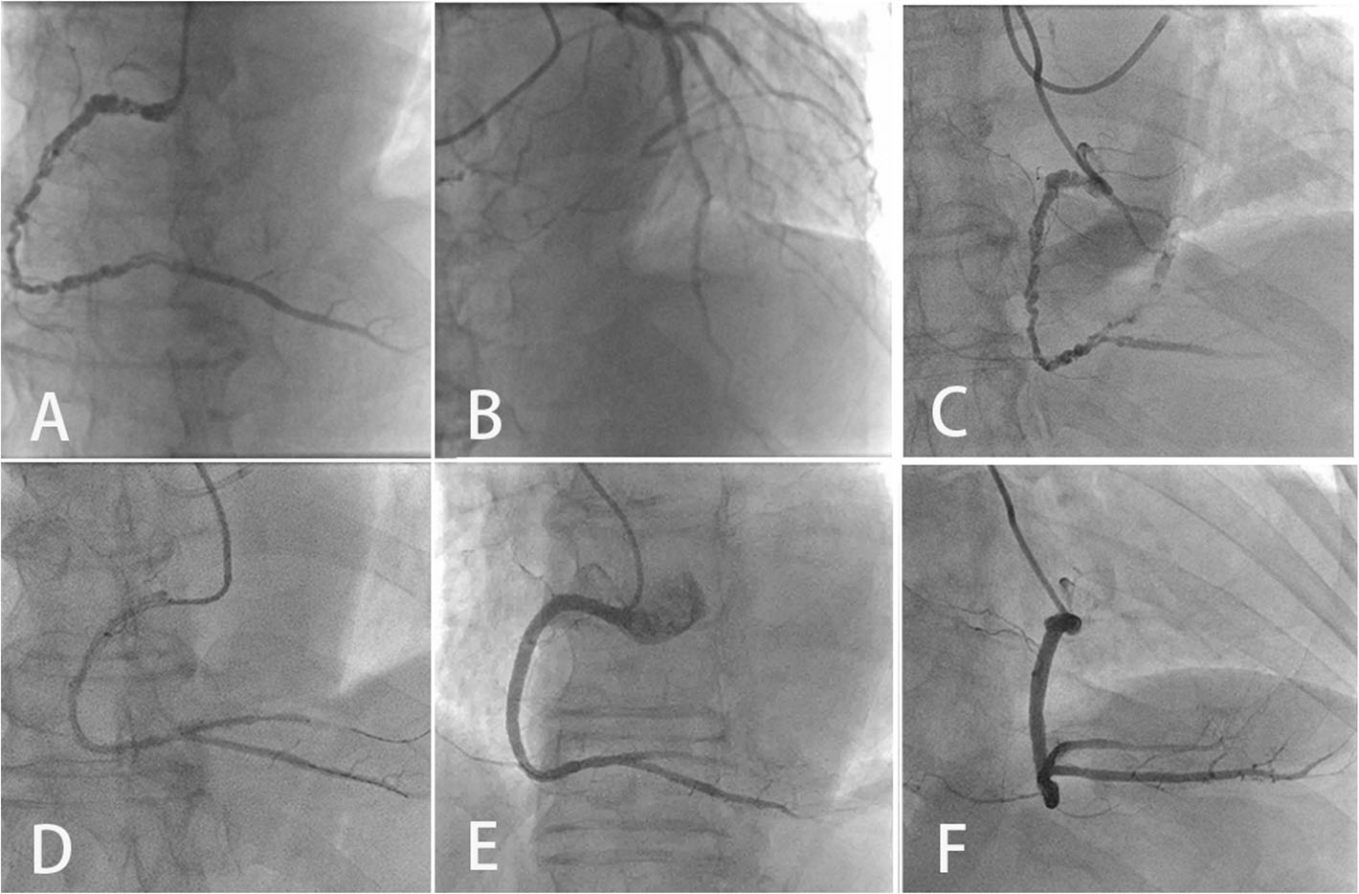
The CAG and PCI data on 12th July 2012. multiple, twisting tiny channels were found in his RCA, which reconnected distally to PDA (**a**). no significant lesions were found in LCA, which providing collaterals to PLB (**b**). Bilateral angiogram showed woven-like changes of RCA (**c**). ‘T’-stent and kissing balloon technique were performed (**d**). Final result showed TIMI 3 flow to PDA vessels (**e**-**f**)

**Fig. 3:**
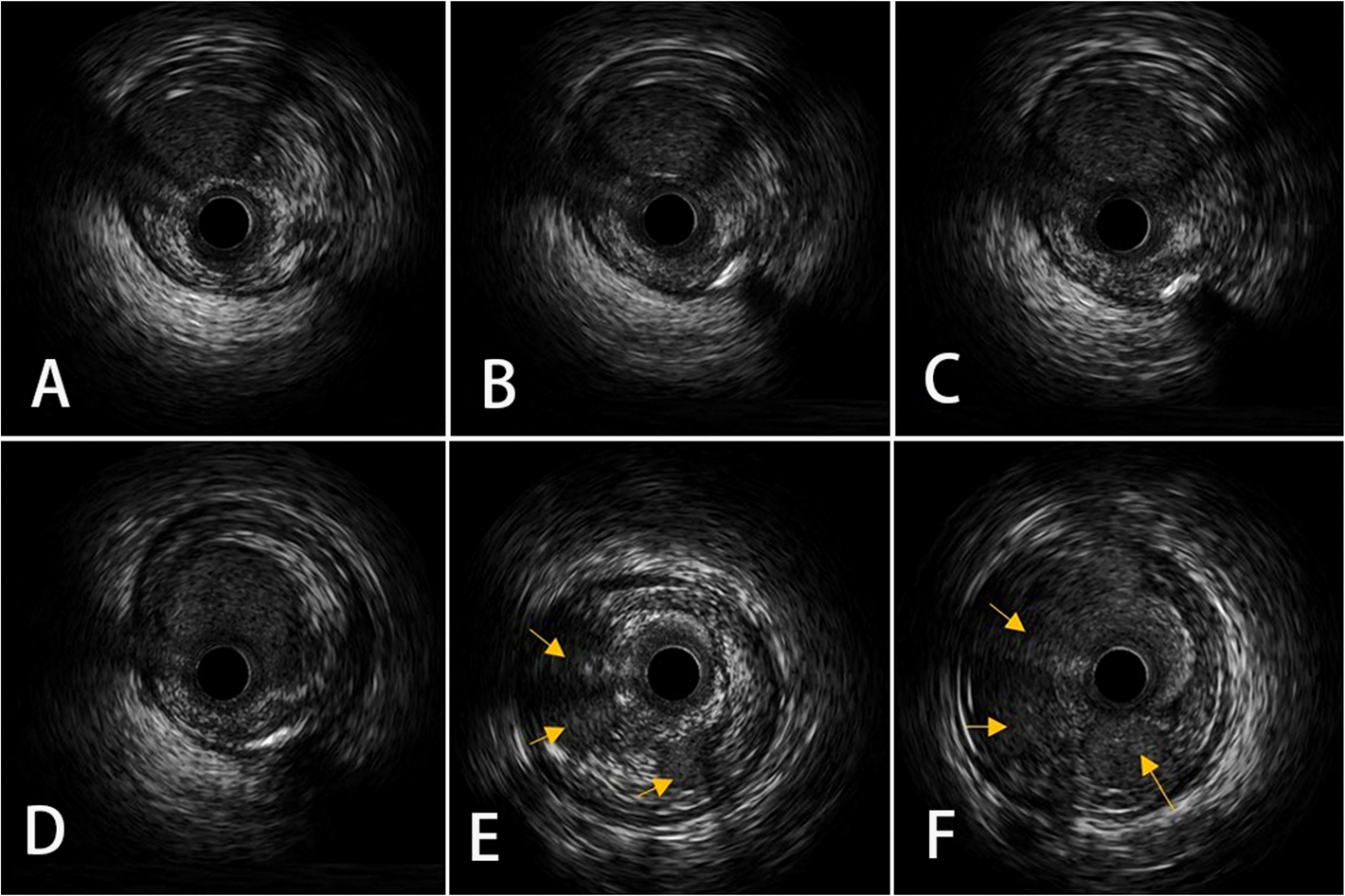
The IVUS finding. IVUS examination found multiple recanalized RCA lumen from distal to proximal, and fusing into one lumen in the ostial segment (**a**-**b**). IVUS pull-back from distal to proximal, dual-lumen from distal merged into one lumen (**c**-**d**). Multiple tiny lumen (yellow arrow) (**e**-**f**) were presented in middle segment, and merged proximally. Intact media were found in from distal to proximal by IVUS

Via bilateral radial artery access was selected for contralateral angiogram (Fig. 2 c) (Additional file 1). Sion and Fielder-XT wires failed in crossing the RCA recanalized segment. With microcatheter supporting, stiffer GAIA second wire was successful in penetrating through the long lesion and reached the distal PDA lumen. As ultrasonic catheter failed in crossing the woven-like lesion at beginning, the lesion was pre-dilated by 2.0 mm*20 mm balloon and then the IVUS examination was implemented successfully. Multiple thrombotic recanalized lumen in RCA was demonstrated in IVUS findings, and the position of guide wire was in the true lumen of RCA (Fig. 3).

After pre-dilation, the flow of second PDA was shown in angiogram. With the support of double-lumen catheter, a Pilot 150 wire was used to penetrate and reached the distal lumen of second PDA. Dilation of both first and second PDA vessels was performed, followed by stent implantation. ‘T’-stent technique was performed in the bifurcation site of distal RCA using the second PDA vessel as branch, and followed by 4 stents (36 mm, 33 mm, 28 mm, 28 mm) overlapping from the proximal of first PDA to the ostium of RCA(Fig. 2 D). After kissing balloon inflation in distal bifurcation site and post-dilation of RCA main trunk, the final angiogram showed restored TIMI 3 flow to PDS vessels (Fig. 2 e-f) (Additional file 2). However, the stent-jailed PLB vessel was still occluded and supplied by contralateral collaterals. Considering the procedure time (almost 2 h) and contrast consumption(350 ml), we stopped the procedure after restoring the PDA flow. A staged PCI to open the PLB vessel is based on the degree of clinical improvement.

The patient recovered well and discharged 6 days after PCI with clopidogrel (75 mg q d), rivaroxaban (2.5 mg bid) and metoprolol (47.5 mg q d). During 9-months clinical follow-up, the patient had no angina episode, the echocardiography showed systolic function of inferior wall was improved and LVEF was 44%.

## Discussion and conclusions

A woven coronary artery is characterized by branching the coronary artery into several smaller vessels, which reconnect distally after twisting along the coronary axis with normal blood flow. As there are very few numbers of woven coronary artery cases, the etiology of this anomaly is not fully explained yet. One case report examining the histopathology of woven coronary artery showed that these lumina shared a coat of elastic tunica and each of them had endothelial coat [2]. Because most cases showed none malignant clinical outcome and the youngest patient was a 9-month-old infant with Kawasaki disease [3], this anomaly is considered to be a rare benign congenital malformation with no treatment needed [4]. However, it has been reported to be associated with thrombus and myocardial infarction [5]. PCI of a local or short lesion with woven change has been reported in few publications [6]. Most cases of woven coronary artery were noticed accidentally during coronary angiography, and now the diagnostic criteria is based on angiographic images. It was mentioned that the woven-like angiographic characteristic might be imitated by thrombotic recanalization. However, because of the lack of prior CAG data and intracoronary image, none detailed case has been reported.

Thrombotic recanalization is an entity occurs after thrombotic occlusion. It is rarely recognized in clinical practice and often misdiagnosed because the angiographic image has nonspecific characteristic. As angiographic findings such as irregular filling defects and intraluminal haziness are not specific, there is no angiographic uniform definition of intracoronary thrombotic recanalization. Intravascular imaging and prior angiographic image should be helpful for the diagnosis.

Most cases of thrombotic recanalization show it happens in a local or short lesion. Our case reported an extremely long segment of woven-like coronary change derived from a previous coronary thrombus event. Differential diagnosis of such woven-like images has significant therapeutic consequence. As the lumina of woven coronary artery has endothelial coat which could be identified by Intravascular imaging, Intravascular ultrasound (IVUS) and Optical coherence tomography (OCT) had been reported to be helpful in differentiating thrombus, dissection or woven structures [7]. OCT has a much higher image resolution than IVUS, so it may provide more details on the characteristics of the thrombi. Because of the complex structure, it may be tough for the catheter of IVUS or OCT to cross over the woven-like lesion. In our case, it was even impossible for the soft guide wire to go through the long channel. The patient’s previous angiogram and history indicated there was a shot thrombotic lesion in his RCA 6 years before. We surmised that the woven-like change was caused by recanalized thrombus and decided to use stiff wire to penetrate through the long structure. The IVUS, the angiogram after PCI, and the clinical outcome confirmed our estimate. In this case, both baseline angiogram and intracoronary image of follow-up were collected. For this patient, PCI could be a better treatment strategy. The detailed history collection, long time followed-up and repeated imaging data including angiogram and intracoronary image should be emphasized in future clinical practice for woven-like coronary change.

## Supplementary information

**Additional file 1.** The angiogram of RCA before PCI. Contralateral angiogram showed the woven-like change in RCA and the PLB was chronic total occlusion.

**Additional file 2.** The angiogram of RCA after PCI. Final result showed that the side-branches were still intact.

## Data Availability

The datasets used in the case are available from the corresponding author upon reasonable request.

## Abbreviations

CAG: Coronary angiography
IVUS: Intravascular ultrasound
LCA: Left coronary artery
LVEF: Left ventricular ejection fraction
OCT: Optical coherence tomography
PCI: Percutaneous coronary intervention
PDA: Posterior descending artery
PLB: Posterior lateral branch
RCA: Right coronary artery

## References

[1] Sane DC, Vidaillet JH (1988). Woven right coronary artery: a previous undescribed congenital anomaly. Am J Cardiol.

[2] Abaci A, Gonul II, Ozkan S, Cemri M (2013). Pathological examination of the woven coronary anomaly. Eur Heart J.

[3] Yıldırım A, Oğuz D, Olguntürk R (2010). Woven right and Aneurysmatic left coronary artery associated with Kawasaki disease in a 9-month-old patient. Cardiol Young.

[4] Rapp AH, Hillis LD (2001). Clinical consequences of anomalous coronary arteries. Coron Artery Dis.

[5] Ayhan S, Ozturk S, Tekelioglu UY, Ocak T (2013). Woven coronary artery anomaly associated with acute coronary syndrome. Int J Angiol.

[6] Joseph SC, D'Antoni AV, Tubbs RS, Gielecki J, Loukas M (2016). Woven coronary arteries: a detailed review. Clin Anat.

[7] Uribarri A, Sanz-Ruiz R, Elizaga J, Fernandez-Aviles F (2013). Pathological insights of a woven coronary artery with optical coherence tomography. Eur Heart J.

